# Intelligent identification of medical and veterinary intracellular protozoa by using self-supervised learning

**DOI:** 10.1186/s13071-026-07257-9

**Published:** 2026-02-09

**Authors:** Veerayuth Kittichai, Morakot Kaewthamasorn, Naruchit Soiphet, Teerawat Tongloy, Santhad Chuwongin, Siridech Boonsang

**Affiliations:** 1 Faculty of Medicine, King Mongkut’s Institute of Technology Ladkrabang, Bangkok, Thailand; 2 Center of Excellence in Veterinary Parasitology, Faculty of Veterinary Science, Chulalongkorn University, Bangkok, 10330 Thailand; 3 School of Integrated Innovative Technology, King Mongkut’s Institute of Technology Ladkrabang, Bangkok, Thailand; 4 Department of Electrical Engineering, School of Engineering, King Mongkut’s Institute of Technology Ladkrabang, Bangkok, Thailand

**Keywords:** Zoonotic diseases, Microscopic examination, Supervised learning, Self-supervised learning, Data partition

## Abstract

**Background:**

Zoonotic diseases pose a major threat to both human and animal health, contributing significantly to global morbidity and mortality. Accurate diagnosis is crucial for effective control and treatment, with microscopic examination serving as the gold standard, supplemented by highly sensitive molecular biology techniques. However, these confirmatory methods require skilled personnel and are subject to inter- and intra-rater variability. An innovative solution lies in artificial intelligence (AI)-powered automated tools, which offer a promising alternative. This study aimed to develop a self-supervised learning (SSL) approach using the Distillation with No Labels (DiNOv2) algorithm to extract features of protozoa from Giemsa-stained blood samples.

**Methods:**

The development of self-supervised learning algorithms, including DiNOv2, was based on archived samples of clinically significant and significant veterinary microorganisms. These models were evaluated in comparison to a baseline Vision Transformer (ViT).

**Results:**

Among the tested SSL models, the DiNOv2-Small version achieved exceptional performance, surpassing 99% accuracy and specificity while maintaining the lowest misclassification rate (0.263). It also demonstrated a high area under the curve (AUC) value of 0.990, underscoring its robust classification capability. Remarkably, even when trained on only 20% of the dataset, the SSL models retained performance levels comparable to baseline models trained on the full dataset. However, further reducing the sample size below 20% led to notable declines in evaluation metrics: accuracy dropped by 5–7%, recall decreased from 37.9% to 35.1%, precision fell from 35% to 25.2%, and the F1 score declined from 31.1% to 21.5%. Additionally, the AUC decreased by 4–11%, while the misclassification rate increased, indicating reduced robustness. A key limitation of this study was the highly imbalanced fine-tuned dataset between classes. Nevertheless, the inconsistent performance observed may be mitigated by employing the larger DiNOv2 model, which improves the F1 score and enhances the model’s ability to handle imbalanced data while reducing reliance on labeled data.

**Conclusions:**

The proposed method can assist laboratory technicians, particularly in resource-limited healthcare settings. Furthermore, the findings support the potential deployment of this AI-based tool for automated screening in both medical and veterinary applications.

**Graphical Abstract:**

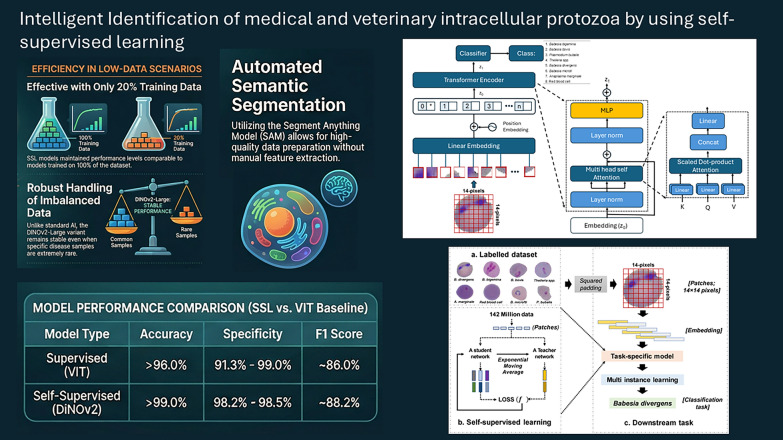

**Supplementary Information:**

The online version contains supplementary material available at 10.1186/s13071-026-07257-9.

## Background

Zoonotic diseases, which transmit between animals and humans, represent a major global public health concern. According to the World Health Organization (WHO), protozoan infections are among the leading causes of human mortality [1]. Important zoonotic protozoa include *Babesia* and *Plasmodium*, with babesiosis—clinically similar to malaria—primarily affecting domestic and wild animals, though human cases also occur. While microscopic examination remains the gold standard for diagnosis and plays a crucial role in disease surveillance and mass drug administration, it faces challenges such as inter- and intra-observer variability and inconsistent reporting quality [2]. Microscopy is not only time-consuming and unsuitable for high-throughput diagnostics, but it is also often inaccessible in remote rural settings—such as peripheral medical clinics lacking electricity and essential healthcare infrastructure—where trained microscopists are typically unavailable [3]. Molecular techniques, though highly sensitive for detecting microbial genetic material [4–6], are limited by high costs, complex sample preparation, and time-consuming procedures. Given the persistent threat of emerging zoonoses, there is an urgent need for innovative diagnostic tools to enhance disease control, improve frontline screening, and support effective treatment strategies [7].

Artificial intelligence (AI) has emerged as a transformative technology, enabled by advancements in high-performance computing and graphics processing units (GPUs), which allow users to efficiently handle large-scale biological datasets. Within AI, machine learning (ML) relies on feature extraction and classification, while deep learning (DL)—a subset of ML—integrates these processes within unified algorithms. DL has been widely adopted across various biomedical applications [2, 8, 9]. Advanced image segmentation techniques are often employed in biological data analysis to precisely identify regions of interest, requiring detailed knowledge of anatomical contours [10]. Segmented images are particularly effective for subsequent classification tasks in both supervised learning (SL) and self-supervised learning (SSL) frameworks. A key advantage of SSL is its ability to minimize reliance on labeled data by leveraging unlabeled datasets while still enhancing model performance—even with limited sample sizes in downstream tasks [11, 12].

Several self-supervised learning (SSL) models have demonstrated significant promise in biomedical applications, particularly in addressing the challenge of limited labeled data. Notable examples include the DINO-ViT model [13], Distillation with No Labels (DiNOv2) model [12], DINO, Masked Autoencoders (MAE), SimCLR [14], and domain-adapted variants such as Surgical-DINO for endoscopic depth estimation [15]. The success of these models underscores their potential to transform biomedical research and healthcare, particularly in scenarios where labeled data are scarce or costly to obtain. Future validation and implementation efforts should focus on further optimizing domain-specific adaptations (e.g., Surgical-DINO’s LoRA layers 4) and expanding their use in multimodal biomedical workflows [15]. Among these, the DINO family of models—including its optimized versions (e.g., DINOv2’s ViT-Small, ViT-Base, and ViT-Large architectures)—has emerged as particularly powerful, offering robust feature extraction capabilities without requiring labels.

This study focuses on developing self-supervised learning (SSL) models using DiNOv2 to extract meaningful features from representative zoonotic protozoa and bacteria—pathogens critically important in human–animal disease transmission. We evaluate the performance of our trained SSL models against supervised learning (SL) counterparts, specifically Vision Transformers (ViT). To assess robustness, we systematically examine the impact of limited training data, testing model performance with progressively reduced datasets (from 80% down to 10% of full data).

Our research makes three key contributions: (1) addressing limitations of SSL models in healthcare applications through knowledge distillation from unlabeled data; (2) demonstrating SSL’s superior feature representation for semantic segmentation without data augmentation, even with highly imbalanced datasets compared with SL approaches; and (3) showing the model’s transfer learning capability to unseen datasets during fine-tuning, despite their exclusion from SSL pretraining.

## Methods

### Sample collection and dataset preparation

Biological samples used in this study were collected as part of a previously described surveillance program targeting vector-borne pathogens [16–18]. Infection status was confirmed using polymerase chain reaction (PCR) followed by Sanger sequencing. The study also included Giemsa-stained smears containing protozoan pathogens such as *Babesia bigemina* (BBI), *Babesia bovis* (BBO), *Theileria* spp., and *Plasmodium bubalis* (water buffalo malaria), as detailed in Table 1. Experienced microscopists examined these stained slides to characterize parasite morphology and developmental stages.

**Table 1:** Sample collection for training the SL and SSL models

Class name	Train						Test	Per-class total	Bio-source	Image source
	Whole; 100%	Partition								
		80%	50%	30%	20%	10%				
1. *Babesia bigemina*	100	80	50	30	20	10	50	150	Goat [22]	Our study
2. *Babesia bovis*	150	120	75	45	30	15	50	200	Cattle [4]	Our study
3. *Plasmodium bubalis*	138	110	69	42	27	14	30	168	Buffalo [5]	Our study
4. *Theileria* spp.	150	120	75	30	20	10	50	200	Buffalo [5]	Our study
5. *Babesia divergens*	20	16	10	10	10	10	8	28	Human	Public
6. *Babesia microti*	10	10	10	10	10	10	9	19	Human	Public
7. *Anaplasma marginale*	1000	800	500	300	200	100	167	1167	Buffalo	Previous work [19]
8. Red blood cell	800	640	400	240	160	80	158	958	Buffalo	Previous work [19]
Total = 2920	2368	—	—	—	—	—	552	—	—	—

*Babesia divergens* and *Babesia microti* are obtained from public resources with url: https://universe.roboflow.com/ceniza/babesia-parasite-mvfql

The dataset was carefully partitioned to avoid selection bias. For our own Giemsa-stained slides (including *Babesia bigemina*, *Babesia bovis*, *Theileria* spp., and *Plasmodium bubalis* [water buffalo malaria]), the physical samples were split for training and testing before any images were captured. For data obtained from public sources (*B. divergens*, *B. microti*, *A. marginale* (AMA), red blood cell [RBC]), we ensured that the training and test sets contained entirely different sources. Microscopic images were captured at 1000× magnification using an oil-immersion light microscope (Olympus CX31, Japan) equipped with a digital camera (Olympus DP21-SAL) at Chulalongkorn University’s Faculty of Veterinary Science. Images were focused on monolayer areas for optimal clarity. The dataset was supplemented with two human *Babesia* species (*B. divergens* and *B. microti*) from RoboFlow’s public repository (https://universe.roboflow.com/ceniza/babesia-parasite-mvfql) and *Anaplasma marginale* images from previous studies [5, 19].

### SAM-based segmentation for dataset preparation

All microscopic images, including both original and publicly sourced data, underwent semantic segmentation using the concatenated Segment Anything Model (SAM) [10, 20, 21]. This transformer-based deep learning model, pretrained on over 1 billion segmentation masks, demonstrates exceptional zero-shot transfer capability without requiring task-specific pretrained weights [10]. Individual cell segmentation was achieved through manual bounding box selection, yielding complete cellular outlines.

#### Complete SAM model specifications

*Model identification*: (1) model name: Segment Anything Model (SAM) with ViT-H backbone—Checkpoint file: sam_vit_h_4b8939.pth; (2) file size: 2.56 GB (636 million parameters); (3) developer: Meta AI Research—official download URL: https://dl.fbaipublicfiles.com/segment_anything/sam_vit_h_4b8939.pth.

*Architecture specifications*: (1) image encoder: ViT-Huge with 16 × 16 patch size, 636 million parameters—input image size: 1024 × 1024 × 3 (RGB); (2) prompt encoder: handles bounding box prompts, converts to 256-dim embeddings; (3) mask decoder: lightweight transformer generating binary segmentation masks; (4) output: three candidate masks per prompt with Intersection over Union (IoU) confidence scores.

Microscope fields were carefully selected on the basis of quality criteria including monolayer regions with minimal cell overlap, in-focus images, representative parasitemia, and adequate Giemsa staining. We captured 5–10 fields per slide, with typically 5–15 viable cells segmented per field.

For bounding box prompt creation, a single trained microscopist (V.K.) manually drew axis-aligned rectangles encompassing each entire cell with a 5–10 pixel buffer beyond the visible cell boundary to ensure complete cell capture. Each bounding box was aligned with the image coordinate system and required approximately 5–10 s to create.

#### Detailed segmentation protocol

For image resolution input to SAM, we used full-resolution microscope images (2048 × 1536 pixels from the Olympus DP21-SAL camera). During processing, SAM automatically resized these images to 1024 × 1024 pixels. The output consisted of segmented cells of variable sizes, typically 80–200 pixels in diameter. We did not apply any color or stain normalization—such as histogram matching, Macenko, Reinhard, or Vahadane methods—nor did we use histogram equalization, color jittering, or other augmentations.

The SAM processing pipeline operated in SamPredictor mode using box prompts. For each prompt, the model generated three candidate masks. The mask with the highest Intersection over Union (IoU) score was automatically selected. Pixels with confidence greater than 0.5 were classified as foreground, on the basis of a threshold of 0.5. The output consisted of binary NumPy arrays (H × W, dtype = bool). Masks with IoU scores above 0.7 were accepted automatically, while those below this threshold were manually reviewed.

Postprocessing and quality control involved morphological closing with a 3 × 3 structuring elements to fill small gaps, followed by morphological opening with the same kernel size to remove noise. Small components less than 10 pixels were removed, and every mask was manually reviewed by the operator. Approximately 7% of cells required re-prompting with adjusted bounding boxes, while 1% were excluded after three failed attempts.

For cell extraction and image creation, background pixels (mask = 0) were set to white (255, 255, 255), and each segmented cell was cropped using a tight bounding box with a 2–5 pixel margin around the masked region. Files were saved in PNG format with lossless compression, resulting in individual cell images typically 80–200 × 80–200 pixels in size.

Special cases were handled systematically. Cells with more than 30% overlap were excluded, while moderate overlap (10–30%) was resolved using the watershed algorithm for boundary separation. When multiple parasites of the same species infected a single cell, the entire cell was segmented as one unit and labeled by species. Cell fragments and out-of-focus cells were removed on the basis of sharp edge criteria and completeness.

The dataset construction achieved a 92.7% success rate, with 2920 cells successfully segmented from approximately 3150 attempted cells. First-attempt success occurred in 92% of cases, 7% required re-prompting, and 1% were ultimately excluded. SAM served solely as a preprocessing tool for dataset construction. Once the 2920 segmented cell images were created, SAM was not used further. The DiNOv2 and ViT models were trained directly on these preprocessed images without any involvement of SAM in the training or inference phases.

Each microscope field containing multiple cells was processed in an iterative manner. A bounding box was drawn around each cell, and the Segment Anything Model (SAM) generated one segmentation mask per cell. Each mask was then saved as an individual image file. This workflow produced 2920 segmented cell images, forming the complete dataset. Most of these cells were successfully extracted from monolayer regions identified during microscopic image acquisition. The dataset was divided into a training set of 2368 cells (81.1%) and a test set of 552 cells (18.9%) (Table 1).

Eight classes were assigned with highly imbalanced data, including 150 cells for *B. bigemina*, 200 cells for *B. bovis*, 168 cells for *P. bubalis*, 200 cells for *Theileria* spp., 28 cells for *B. divergens*, 19 cells for *B. microti*, 1167 cells for *A. marginale*, and 958 cells for red blood cells. Both the full training and testing datasets were used to train and evaluate the Vision Transformer (ViT) as a baseline supervised learning (SL) model and DiNOv2 as a self-supervised learning (SSL) model. The dataset was randomly partitioned into training subsets of 80%, 50%, 30%, 20%, and 10% to assess whether the SSL model could effectively learn from different data volumes while maintaining performance comparable to the SL model. Additionally, for classes with smaller sample sizes, at least ten images per class were retained to prevent classification bias in the downstream process of the SSL model.

In this study, no hand-crafted data augmentation was applied. A previous publication suggested that handcrafted photometric augmentations could be optional for effective learning with the DiNOv2 model [23]. This is because shared, crop, and crop + resize augmentations did not appear to influence the learning of invariances compared with the original datasets, such as ImageNet1k, ImageNet22k, and LVD-142 M. Instead, these augmentations primarily impacted model learning by increasing the dataset size.

#### Expert validation process

In the dataset preparation process, all parasite species identifications were performed through a systematic two-stage expert validation workflow. Dr. Arnuphapprasert Apinya (postdoctoral fellow, Faculty of Veterinary Science, Chulalongkorn University; 8 years of experience in veterinary parasitology) performed initial morphological classification of all 2920 cells following standardized diagnostic criteria established by the World Organization for Animal Health (WOAH) and adapted WHO guidelines for blood parasite identification.

Diagnostic criteria included parasite size relative to host erythrocyte, morphological form (piriform, round, or amoeboid), intraerythrocytic location, Giemsa staining characteristics (chromatin and cytoplasm coloration), and developmental stage (ring form, trophozoite, schizont, or merozoite).

Subsequently, Dr. Morakot Kaewthamasorn (associate professor, Faculty of Veterinary Science, Chulalongkorn University; > 10 years of specialized experience in vector-borne protozoan diagnostics) systematically reviewed and validated all initial classifications.

The validation process achieved 92.4% agreement, with 2698 of 2920 cells approved without modification and 222 cells (7.6%) requiring consensus discussion between both experts. Cases flagged during validation were jointly reviewed by both experts using detailed morphological examination and, when available, preliminary molecular testing results.

Per-class approval rates ranged from 85.2% to 98.7%, with highest agreement for morphologically distinct and abundant classes (red blood cell [RBC]: 98.7%; *A. marginale*: 96.3%) and lower agreement for morphologically similar species pairs requiring detailed examination (*B. bigemina* versus *B. bovis*: 88.9%; *B. divergens* versus *B. microti*: 85.2%).

### Model architectures

#### Baseline as vision transformer model

**Fig. 1:**
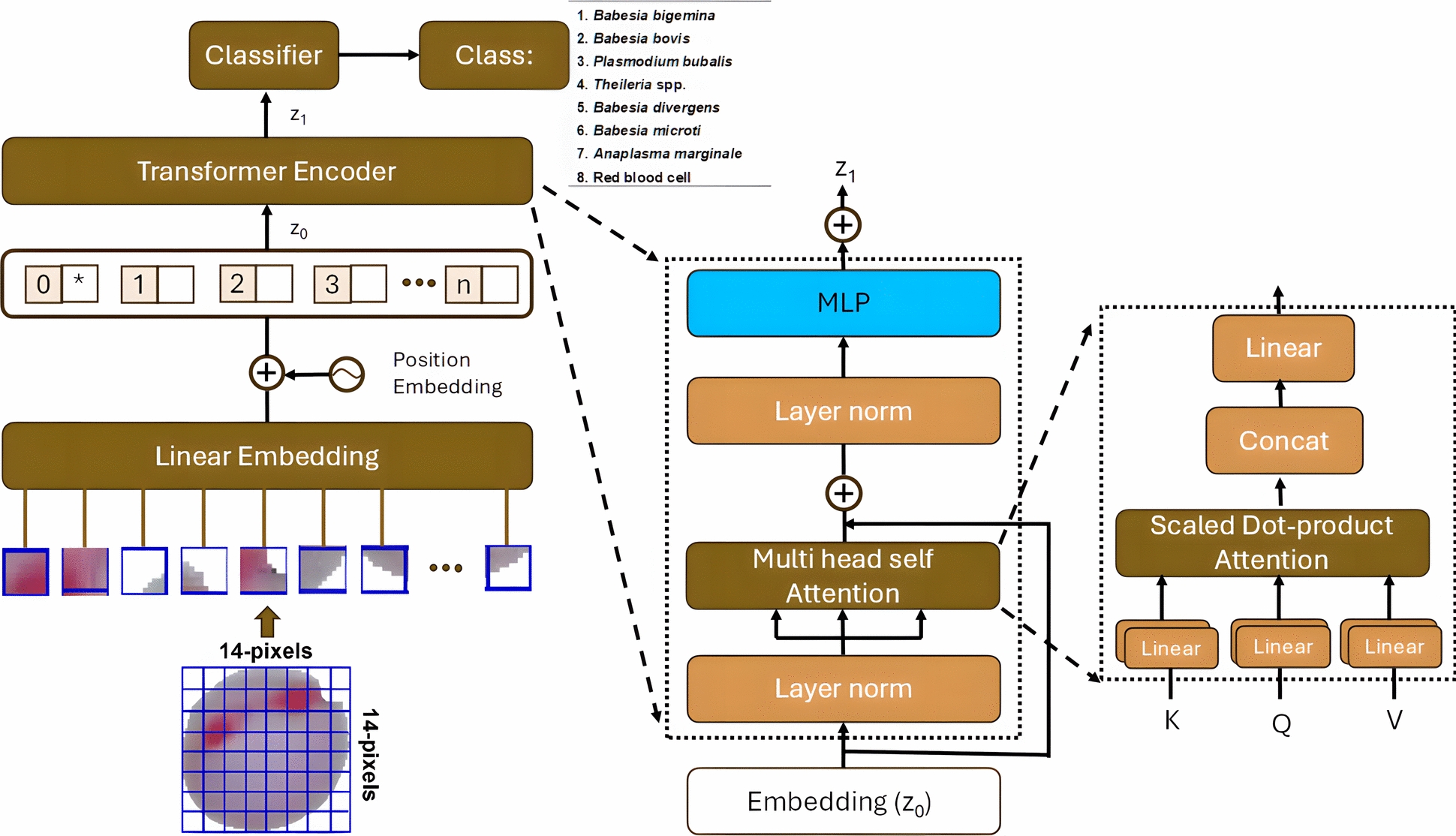
Architecture of Vision Transformer (ViT) algorithm

The adaptation of the transformer model, known as the Vision Transformer (ViT), is designed for image recognition by processing images as multiple patches, similar to word tokens in natural language processing (Fig. 1). The Vision Transformer is widely used across various domains, including medical research [24–26]. The architecture of the proposed model consists of four main components. First, the patch embedding layer divides input images into 16 × 16 patches and projects them as embeddings. Next, position embeddings are added to incorporate spatial information. The transformer encoder then applies self-attention mechanisms to process the patch embeddings. Finally, the classification head predicts the class labels. The scaled dot-product attention is formulated as follows (Eq. 1):

1 $$Attention \left(Q, K, V\right)=softmax (\frac{{QK}^{T}}{\sqrt{{d}_{k}}})V$$

where *Q* is Query matrix (dimensions: *n* × *d*_*k*_) representing input features transformed for matching; *K* is the key matrix (dimensions: *m* × *d*_*k*_) representing stored information for lookup; *V* is the value matrix (dimensions: *m* × *d*_*v*_) containing actual information to retrieve; d_k_ is the dimension of key/query vectors, used as scaling factor to prevent gradient vanishing; d_v_ is the dimension of value vectors, determining the output embedding dimension; *n* is the number of query positions; *m* is the number of key/value positions; and*softmax* is the normalization function converting attention scores to probability distribution. The scaling by √*d*_*k*_ prevents dot products from growing too large in high dimensions. The SoftMax equation was used for normalizing the elements within the vector [27].

### Self-supervised learning model

Distillation with No Labels (DiNOv2) is a cutting-edge self-supervised learning algorithm that leverages Vision Transformers (ViTs) as its backbone and integrates self-distillation with multiobjective training [28]. This approach minimizes the reliance on extensive ground truth labels and human-annotated label quality, reducing the need for large training datasets when fine-tuning the model for specific downstream tasks. This efficiency highlights the rationale for the widespread adoption of self-supervised learning (SSL) models in medical applications [11–13].

Four model variants are proposed:

(i) Distilled lightweight (ViT-S/14 (Distilled)) with 21 million parameters, processing 384 embedding dimensions.
(ii) Balanced performance (ViT-B/14 (Distilled)) with 86 million parameters, processing 768 embedding dimensions.
(iii) High performance (ViT-L/14 (Distilled)) with 300 million parameters, processing 1024 embedding dimensions.
(iv) Largest (ViT-g/14), featuring 1.1 billion parameters and processing 1536 embedding dimensions.

**Fig. 2:**
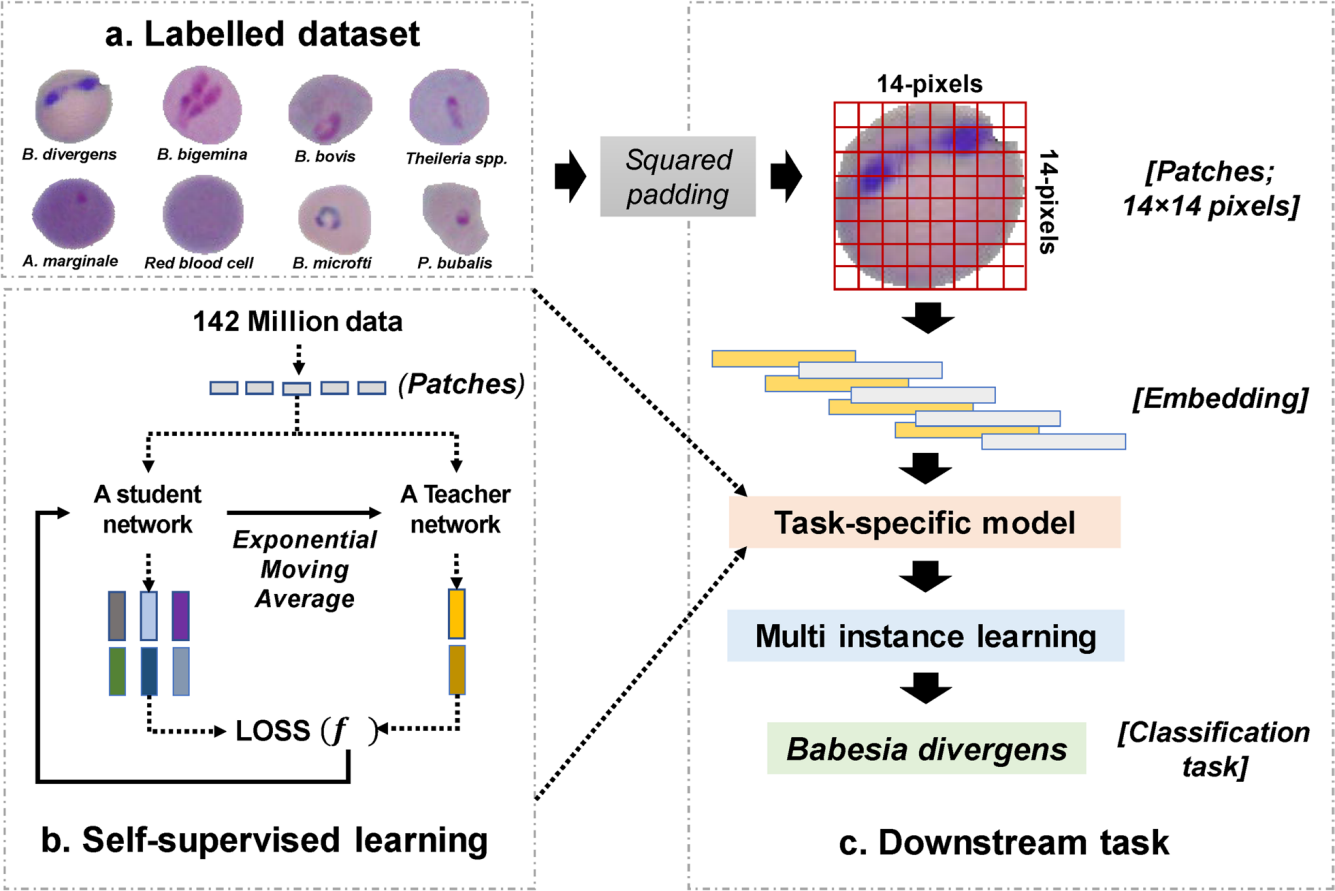
Model architecture. The workflow is comprised of (**a**) the labeled dataset for eight-class names for segmented cells and (**b**) the self-supervised learning by distilling knowledge output between the student- and teacher networks using the cross-entropy loss. The exponential moving average (EMA) is for updating the teacher network, (**c**) the downstream task that is for fine tuning the task specific process

All models are trained on a structured dataset comprising 142 million images (LVD-142 M) (Fig. 2a–c).

The first three models are designed to reduce computational costs compared with the largest model by distilling knowledge from ViT-g/14. Cross-entropy loss was applied between the extracted features of the student and teacher networks, enabling knowledge transfer without the need for labeled data. The teacher network was updated using an exponential moving average (Fig. 2b).

Additionally, a masked cross-entropy loss was employed, where the student network predicted masked random patches and aligned them with the corresponding unmasked patches from the teacher network. The total combined cross-entropy loss is formulated as follows (Eq. 2):

2 $${L}_{total }= {\lambda }_{1}{L}_{DINO}+ {\lambda }_{2}{L}_{iBOT}+{\lambda }_{3}{L}_{KoLeo}$$

where *L*_*DINO*_ is the image-level cross-entropy loss between student and teacher network outputs, encouraging the student to produce similar global image representations as the teacher, *L*_*iBOT*_ is the image-BERT-like masked modeling loss at patch level, where the student predicts masked patches using unmasked context and the teacher provides target representations for masked regions; and *L*_*KoLeo*_ is the KoLeo regularization term preventing dimensional collapse, ensuring embedding dimensions remain informative and non-redundant—λ₁, λ₂ = loss weighting coefficients (both typically set to 1.0 in original DiNOv2). Teacher network is updated via exponential moving average (EMA) of student weights [29].

### Model training configuration and hyperparameters

#### Vision transformer (baseline supervised learning model)

##### Architecture configuration

The architecture uses three model variants—ViT-Small, ViT-Base, and ViT-Large—each processing input images with dimensions of 224 × 224 × 3 (RGB). These images are divided into patches of 16 × 16 pixels before being processed through the network, which is initialized using ImageNet-21 k pretrained weights. Finally, a classification head consisting of a linear layer maps the resulting embedding dimensions to eight distinct classes.

##### Optimization settings

The models are optimized using AdamW (Adam with decoupled weight decay) with an initial learning rate of 1×10^−4^ and a weight decay of 0.05. The optimizer uses beta coefficients of ${\beta }_{1}=0.9$ and ${\beta }_{2}=0.999$, with an epsilon value of $\epsilon =1\times {10}^{-8}$. To manage the learning rate, a cosine annealing scheduler with linear warmup is employed; this includes a 10-epoch warmup phase where the rate gradually increases from 0 to the initial value. Following this warmup, a cosine decay is applied to transition the learning rate toward a minimum of $1\times {10}^{-6}$.

##### Training protocol

Training is conducted with a batch size of 32 images per batch over a maximum of 300 epochs. To prevent overfitting, an early stopping mechanism is implemented with a patience of 10 epochs on the basis of a training loss plateau. The model is optimized using an unweighted cross-entropy loss function, and gradient clipping is applied with a maximum norm of 1.0 to ensure numerical stability.

#### DiNOv2 (self-supervised learning models)

##### Pretrained model configuration

The models were obtained from the official DiNOv2 GitHub repository. The study uses three specific model variants: DiNOv2-Small (ViT-S/14), with 21 million parameters and 384-dimensional embeddings; DiNOv2-Base (ViT-B/14), with 86 million parameters and 768-dimensional embeddings; and DiNOv2-Large (ViT-L/14) featuring 300 million parameters and 1024-dimensional embeddings. All variants employ a patch size of 14 × 14 pixels and were pretrained on the LVD-142 M dataset, which consists of 142 million curated images. The pretraining method uses self-distillation, specifically incorporating knowledge distillation from a larger ViT-g teacher model.

##### Fine-tuning strategy, optimization settings, and training protocol

The fine-tuning strategy employs full fine-tuning with all backbone layers trainable from the start. The classification head consists of a single linear layer mapping the embedding dimension to eight classes, initialized via Kaiming initialization and preceded by a dropout rate of 0.1. Optimization is handled by AdamW with an initial learning rate of $5\times {10}^{-5}$, weight decay of 0.05, beta coefficients of ${\beta }_{1}=0.9$ and ${\beta }_{2}=0.999$, and an epsilon value of $\epsilon =1\times {10}^{-8}$. A cosine annealing scheduler manages the learning rate with a 5-epoch linear warmup, transitioning to a minimum learning rate of $1\times {10}^{-6}$.

##### Hardware and software environment

The training protocol uses a batch size of 32 images per batch over a maximum of 300 epochs, incorporating early stopping with a patience of 10 epochs and unweighted cross-entropy loss. To optimize performance, mixed precision training (FP16) is used with automatic mixed precision. The hardware environment includes a single NVIDIA A100 GPU (40 GB VRAM), an AMD EPYC 7763 64-Core Processor, and 256 GB of DDR4 RAM. The software stack is built on Python 3.10 and PyTorch version 2.0.1 with CUDA 11.8, leveraging the *timm* library for ViT implementation, torchvision for preprocessing, NumPy and Pandas for data handling, and Scikit-learn for metrics.

Training durations average 4–6 h per Vision Transformer variant and 2–3 h per DiNOv2 variant, totaling approximately 45 GPU-hours. To ensure reproducibility, random seeds of 42, 123, and 456 were used for three independent runs with cuDNN deterministic mode enabled.

##### Image preprocessing pipeline

The image preprocessing pipeline begins with original 2048 × 1536 pixel microscope images captured by an Olympus DP21-SAL camera. SAM segmentation is applied at full resolution to extract cells (typically 80–200 pixels in diameter) as PNG files with transparency. For model input, images are resized to 224 × 224 pixels using bilinear interpolation, preserving aspect ratios with white padding. Pixel values are normalized to a [0, 1] range by dividing by 255.0 within the RGB color space. Notably, this pipeline explicitly excludes color or stain normalization (Macenko, Reinhard, Vahadane), histogram equalization, data augmentation (rotation, flipping, jittering), and test-time augmentation (TTA).

### Experimental design

The experimental setup consisted of three main designs:

(i) Comparison of models: DiNOv2 SSL model variants were compared with ViT model variants, which served as baseline deep learning models. All versions of both SSL and SL models were trained on the full dataset to determine whether the best performing SSL model could surpass the SL models.
(ii) Data partitioning analysis: Based on the performance observed in the previous experiment, the dataset was partitioned into training subsets ranging from 80% to 10% of the full data. This was done to identify the optimal dataset size required for effective SSL model training.
(iii) Class-wise performance assessment: The SSL models trained with different sample sizes were evaluated to examine how varying dataset sizes and image feature variability impact neural network learning and classification performance at the class level.

### Evaluations

The potential performance of both the SL and SSL models was evaluated using various metrics derived from confusion matrix tables [12]. Four key parameters were extracted from the confusion matrix: true positive (TP), which correctly predicts a positive outcome; true negative (TN), which correctly predicts a negative outcome; false negative (FN), where a negative outcome is incorrectly predicted; and false positive (FP), where a positive outcome is incorrectly predicted. Statistical metrics were calculated on the basis of TP, TN, FN, and FP, as detailed in Eqs. (3)–(7).

3 $$Accuracy=\frac{TP+TN}{TP+TN+FP+FN}$$

4 $$Specificity=\frac{TN}{TN+FP}$$

5 $$Recall=\frac{TP}{TP+FN}$$

6 $$Precision=\frac{TP}{TP+FP}$$

7 $$F1 score=2\times \frac{Recall \times Precision}{Recall+ Precision}$$

The area under the receiver operating characteristic curve (AUC-ROC) is a metric used to evaluate a trained model’s ability to classify query images across all threshold values [30]. An ideal classification model achieves an AUC of 1.000, whereas a model performing at random yields an AUC of 0.500 or lower. The area under the precision-recall curve (AUC-PR) evaluates a model’s ability to identify positive cases with high precision and recall, making it the preferred metric for imbalanced datasets. A perfect model scores 1.000, while random guessing scores 0.500. The expected calibration error (ECE) assesses the reliability of a model’s predicted confidence scores. A model is well-calibrated if its confidence aligns with its accuracy (at 95% confidence). An ECE below 0.050 indicates strong calibration.

## Results

**Fig. 3:**
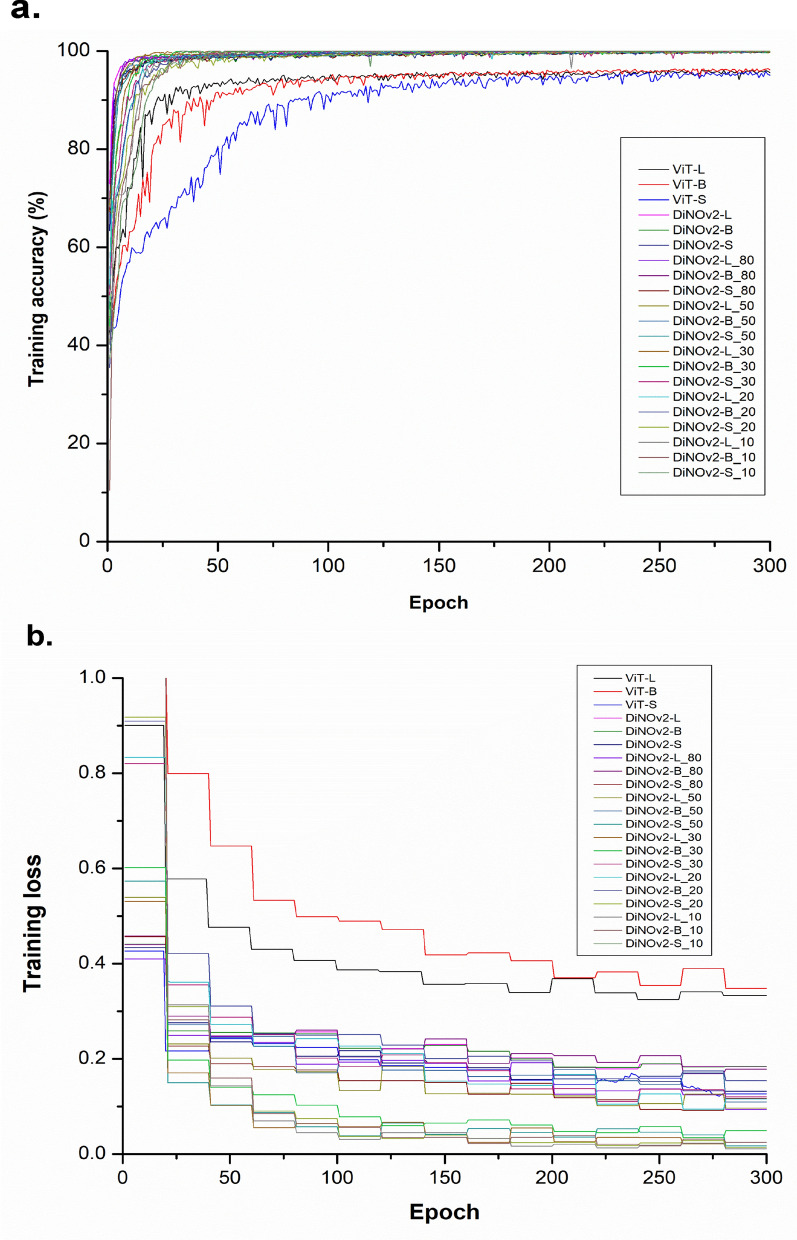
Optimal training condition. The training accuracy and loss are presented in **a** and **b**, respectively. Each plot includes both supervised learning models (ViT versions) and self-supervised learning models (DiNOv2 versions)

This study aimed to detect seven medically and veterinary-relevant microorganisms using advanced self-supervised learning (SSL) algorithms, particularly the DiNOv2 models [12, 31]. The main goal was to develop highly optimized SSL models capable of performing well with limited training data and to evaluate their performance against baseline Vision Transformer (ViT) models [24, 26]. The selected pathogens include two human-infecting *Babesia* species (*B. divergens* and *B. microti*) (Fig. 2), along with two *Babesia* species that infect animals (*B. bigemina* and *B. bovis*), *Theileria* spp., and the bacterial species *A. marginale*. Additionally, *P. bubalis*, an apicomplexan parasite causing water buffalo malaria, was included in the dataset. To reduce false-negative outcomes in model training, animal red blood cells (aRBCs) were used as an outgroup. The top-performing models were chosen for further validation on the basis of training performance plateauing, indicating optimization (Fig. 3).

### Comparative performance of selected models

**Fig. 4:**
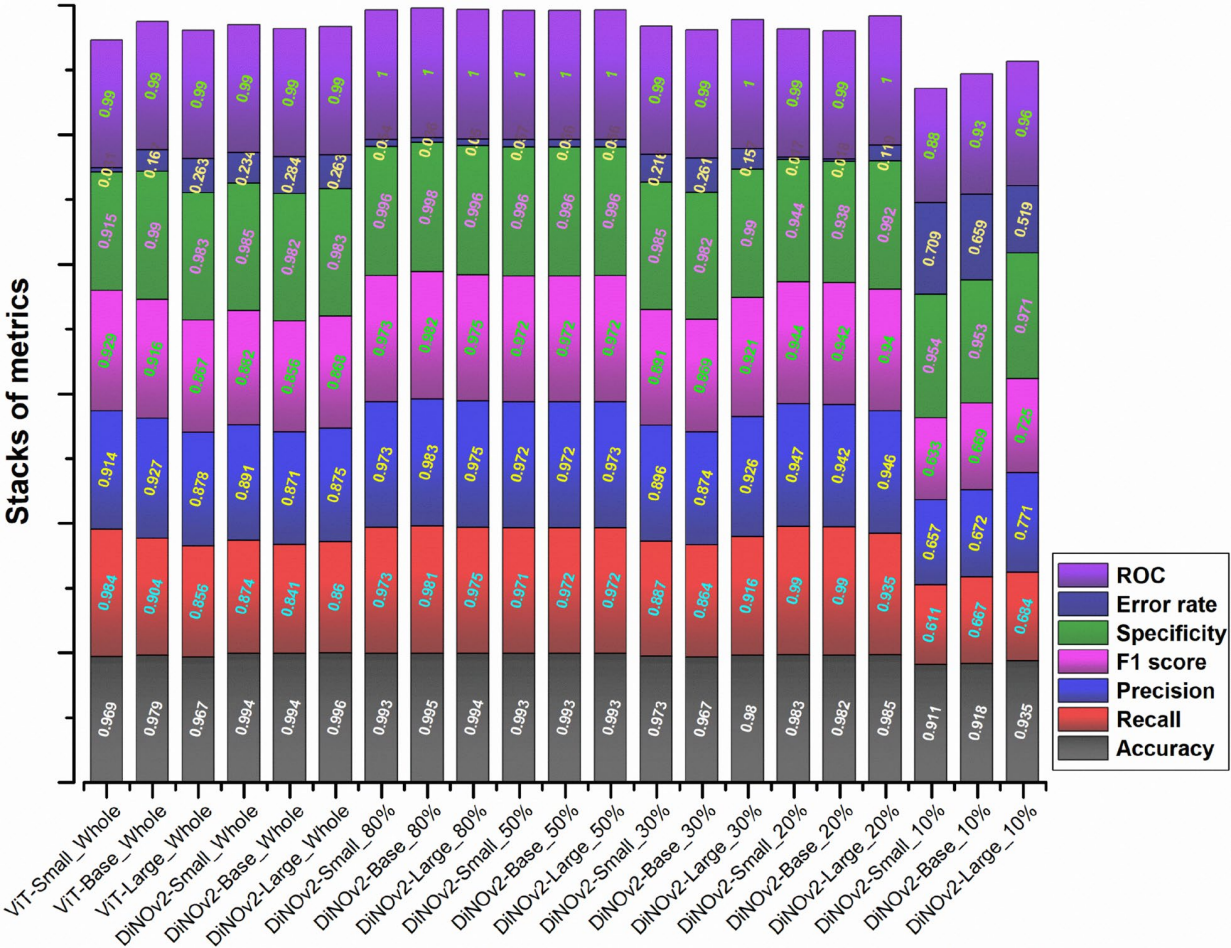
Average performance comparison of trained models

A comprehensive evaluation of model performance was conducted across two distinct experiments, assessing key statistical metrics including accuracy, recall, precision, F1 score, specificity, and receiver operating characteristic (ROC) curve analysis (Fig. 4). Initial analysis of fully trained models on the complete dataset revealed comparable performance between baseline ViT models (ViT-Small, ViT-Base, and ViT-Large) and their DiNOv2 counterparts (Small, Base, and Large). Among the SSL models, DiNOv2-Small achieved the lowest misclassification rate (0.263) and delivered the highest performance in true-positive detection, with a recall of 0.874, precision of 0.891, and an F1 score of 0.882. Additionally, it achieved exceptional accuracy and specificity, both exceeding 99%. The model also demonstrated outstanding discriminative ability, with an AUC-ROC of 0.990, further confirming its robust classification performance.

SSL models were systematically evaluated using progressively smaller training subsets (80%, 50%, 30%, 20%, and 10% of total data) to determine whether their performance could remain competitive with baseline models trained on the full dataset.

Analysis showed that model performance remained stable when training data were reduced to 20% of the full dataset, with both SL and SSL models maintaining comparable metrics to those trained on complete data. However, further reduction to 10% training data resulted in significant performance degradation. Key metrics showed marked declines: accuracy decreased by 5–7 percentage points, recall dropped from 37.9% to 35.1%, precision fell sharply from 35% to 25.2%, and the F1 score declined from 31.1% to 21.5%. This pattern was corroborated by ROC analysis, which revealed a 4–11% reduction in AUC values alongside increased misclassification rates.

Notwithstanding this performance reduction, the DiNOv2-Large model demonstrated superior capability compared with other SSL models at the 10% training data level, attaining 93.5% accuracy, 91.1% specificity, and a 0.960 AUC score. These results indicate that the DiNOv2 architecture maintains robust classification performance even under significant data constraints.

### Class-wise comparison of selected-trained models

**Fig. 5:**
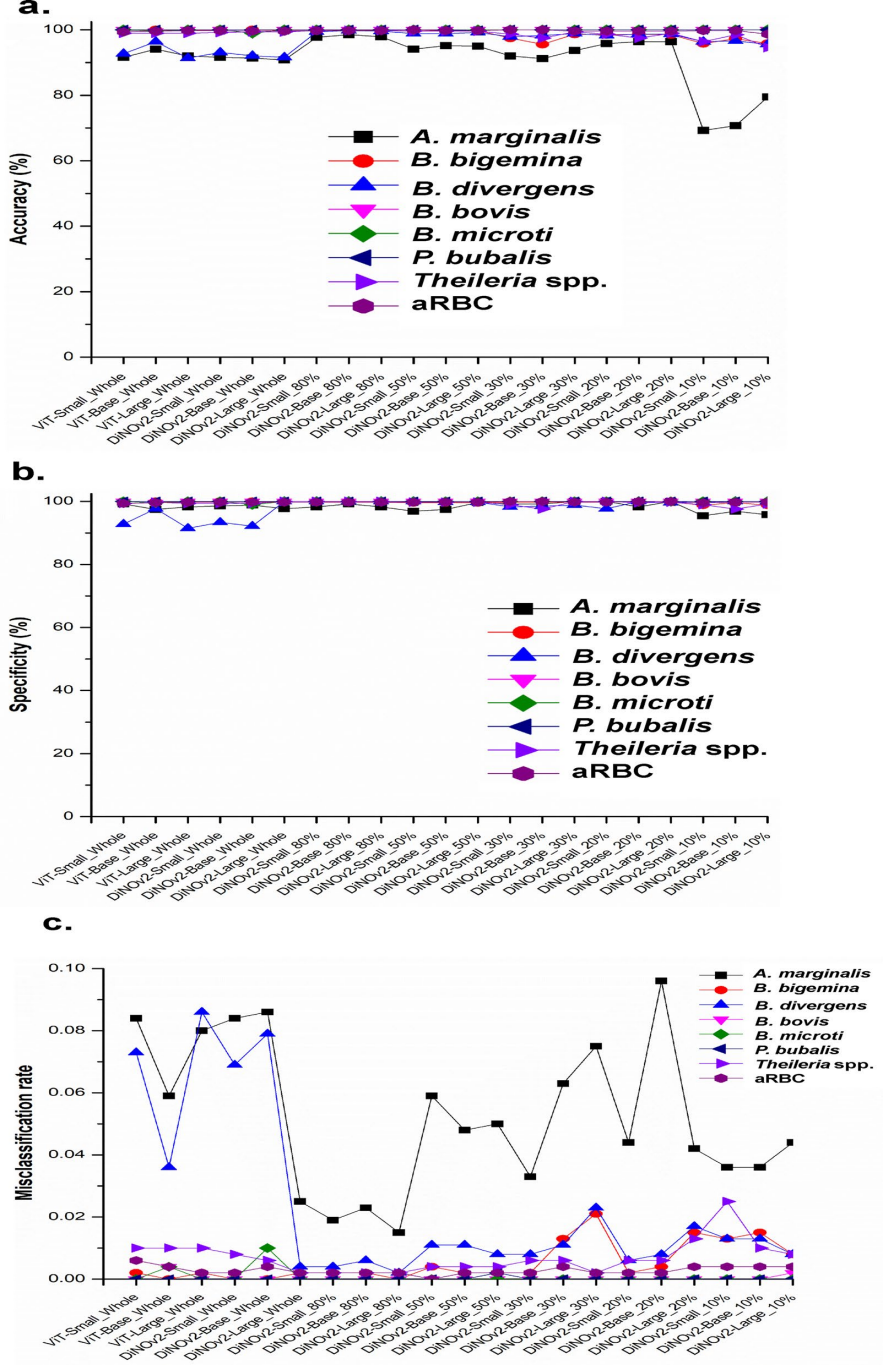
General performance between SSL versus baseline models. The evaluation includes (**a**) accuracy, (**b**) specificity, and (**c**) misclassification rate. The baseline models used are the Small, Base, and Large versions of the Vision Transformer, while the SSL models are the Small, Base, and Large versions of DiNOv2. A vertical dashed line is used to separate the two sides: the left side represents models trained on the full dataset (both SL and SSL), while the right side shows the performance of SSL models trained on data partitions

We evaluated multiclass classification performance across eight distinct categories: *B. bigemina*, *B. divergens*, *B. bovis*, *B. microti*, *A. marginale*, *P. bubalis*, *Theileria* spp., and aRBC. While the misclassification rate showed variability and increased when training with less than 80% of the full dataset, the overall error distribution remained consistent with baseline model patterns (Fig. 5).

However, three classes—*B. bigemina*, *B. divergens*, and *Theileria* spp.—exhibited substantially higher misclassification rates as training data diminished. In contrast, *A. marginale* maintained relatively stable accuracy until the training data were reduced to 20%, at which point its performance sharply declined, leading to a 10% drop in specificity across all classes.

Across all data partition scenarios, the DiNOv2-Large model demonstrated superior performance in all three key evaluation metrics compared with other models. This consistent advantage underscores its robustness for classification tasks, even when trained on substantially reduced datasets relative to baseline models.

This subsection analyzed true-positive (TP)-based metrics—recall, precision, and F1 score. Results revealed that most classes maintained consistent recall values regardless of whether models were trained on full or partitioned datasets (Table 2). However, for *A. marginale* (AMA), both SL and SSL models trained on the full dataset exhibited reduced recall, likely attributable to high dataset variability impairing algorithmic learning.

**Table 2:** Class-wise comparison-based recall

Model version	Data partition			Class name						
		AMA	BBI		BBO	BDI	BMI	PBU	THE	aRBC
ViT-Small	Whole	0.754	0.980		0.920	1.000	1.000	1.000	0.933	1.000
ViT-Base	Whole	0.868	1.000		0.900	1.000	1.000	1.000	0.933	1.000
ViT-Large	Whole	0.784	1.000		0.900	1.000	1.000	1.000	0.967	1.000
DiNOv2-Small	Whole	0.766	1.000		0.920	1.000	1.000	1.000	1.000	0.980
DiNOv2-Base	Whole	0.754	1.000		0.900	1.000	1.000	1.000	0.967	1.000
DiNOv2-Large	Whole	0.743	1.000		0.940	1.000	1.000	1.000	0.967	1.000
DiNOv2-Small	80%	0.964	1.000		0.940	1.000	1.000	1.000	0.967	1.000
DiNOv2-Base	80%	0.970	1.000		0.980	1.000	1.000	1.000	0.967	1.000
DiNOv2-Large	80%	0.964	1.000		0.960	1.000	1.000	1.000	0.967	1.000
DiNOv2-Small	50%	0.976	1.000		0.920	1.000	1.000	1.000	0.967	1.000
DiNOv2-Base	50%	0.976	1.000		0.940	1.000	1.000	1.000	0.933	1.000
DiNOv2-Large	50%	0.964	1.000		0.940	1.000	1.000	1.000	0.933	1.000
DiNOv2-Small	30%	0.766	0.760		0.940	1.000	1.000	1.000	0.933	1.000
DiNOv2-Base	30%	0.802	0.560		0.960	1.000	1.000	1.000	0.967	1.000
DiNOv2-Large	30%	0.898	0.880		0.980	1.000	1.000	1.000	0.900	0.980
DiNOv2-Small	20%	0.964	0.940		0.980	1.000	1.000	1.000	0.967	1.000
DiNOv2-Base	20%	0.976	0.900		0.940	1.000	1.000	1.000	0.967	0.980
DiNOv2-Large	20%	0.958	0.940		0.960	1.000	1.000	1.000	0.933	0.980
DiNOv2-Small	10%	***0.042***	***0.600***		***0.840***	***1.000***	***1.000***	***1.000***	***0.933***	***1.000***
DiNOv2-Base	10%	***0.084***	***0.800***		***0.860***	***1.000***	***1.000***	***1.000***	***0.967***	***0.980***
DiNOv2-Large	10%	***0.359***	***0.560***		***0.860***	***0.875***	***1.000***	***1.000***	***0.967***	***0.980***

The bold and italicized numbers indicate a significantly low performance when using the 10% data partition. The baseline models used are the Small, Base, and Large versions of the Vision Transformer, while the SSL models are the Small, Base, and Large versions of DiNOv2. AMA, *A. marginale*; BBI, *B. bigemina*; BBO, *B. bovis*; BDI, *B. divergens*; BMI, *B. microti*; PBU, *P. bubalis*, THE, *Theileria* spp.; aRBC, animal RBC

**Fig. 6:**
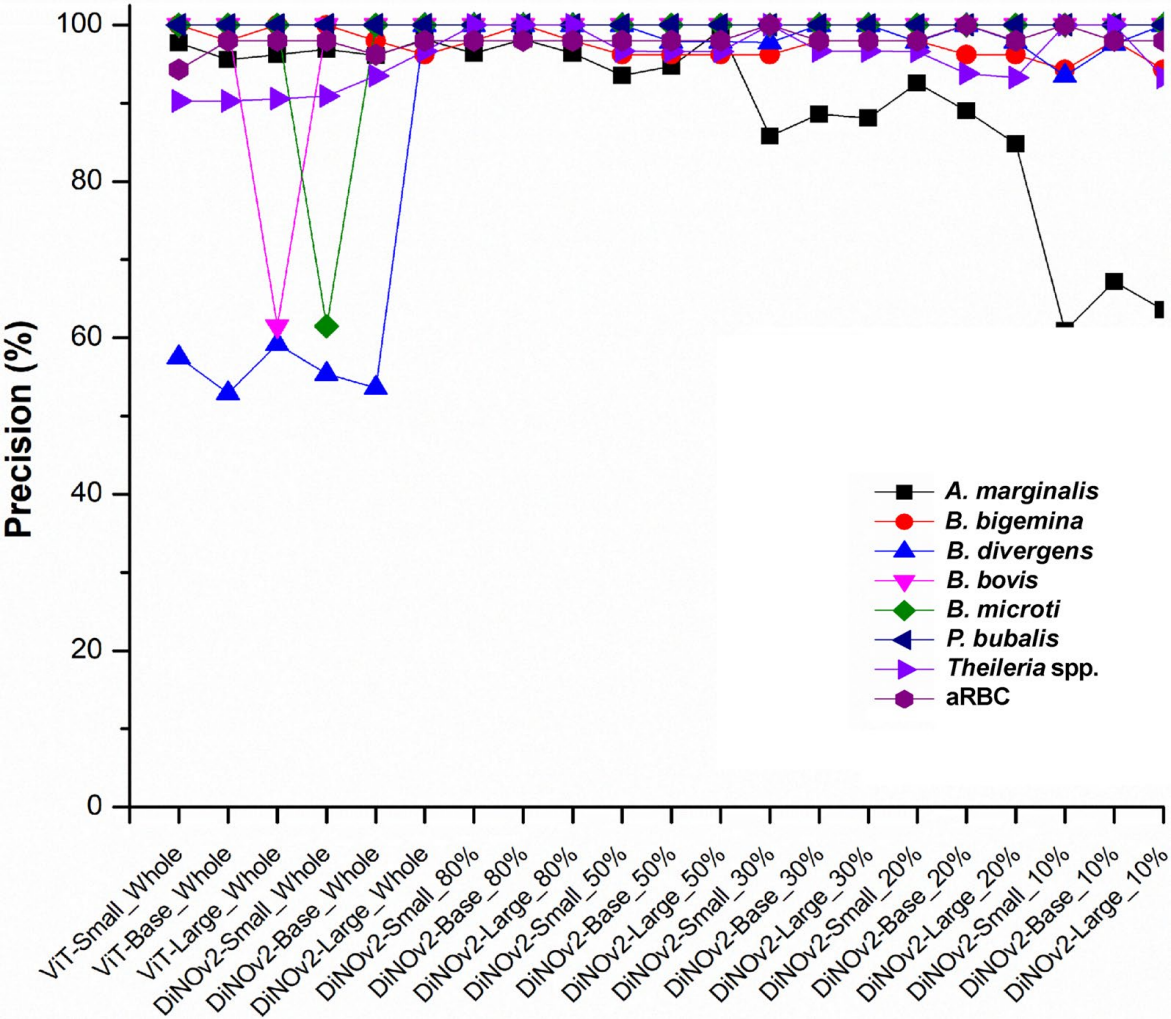
True-positive values based on the precision. The baseline models used are the Small, Base, and Large versions of the Vision Transformer, while the SSL models are the Small, Base, and Large versions of DiNOv2. A vertical dashed line separates the two sides: the left side represents models trained on the full dataset (both SL and SSL), and the right side shows the performance of SSL models trained on data partitions

While model performance declined at the 10% data partition level—most notably for AMA, BBI, and BBO classes—five other classes preserved stable recall values. Precision exhibited comparable patterns to recall, though the AMA class showed deterioration at both 30% and 10% data partitions (Fig. 6).

**Fig. 7:**
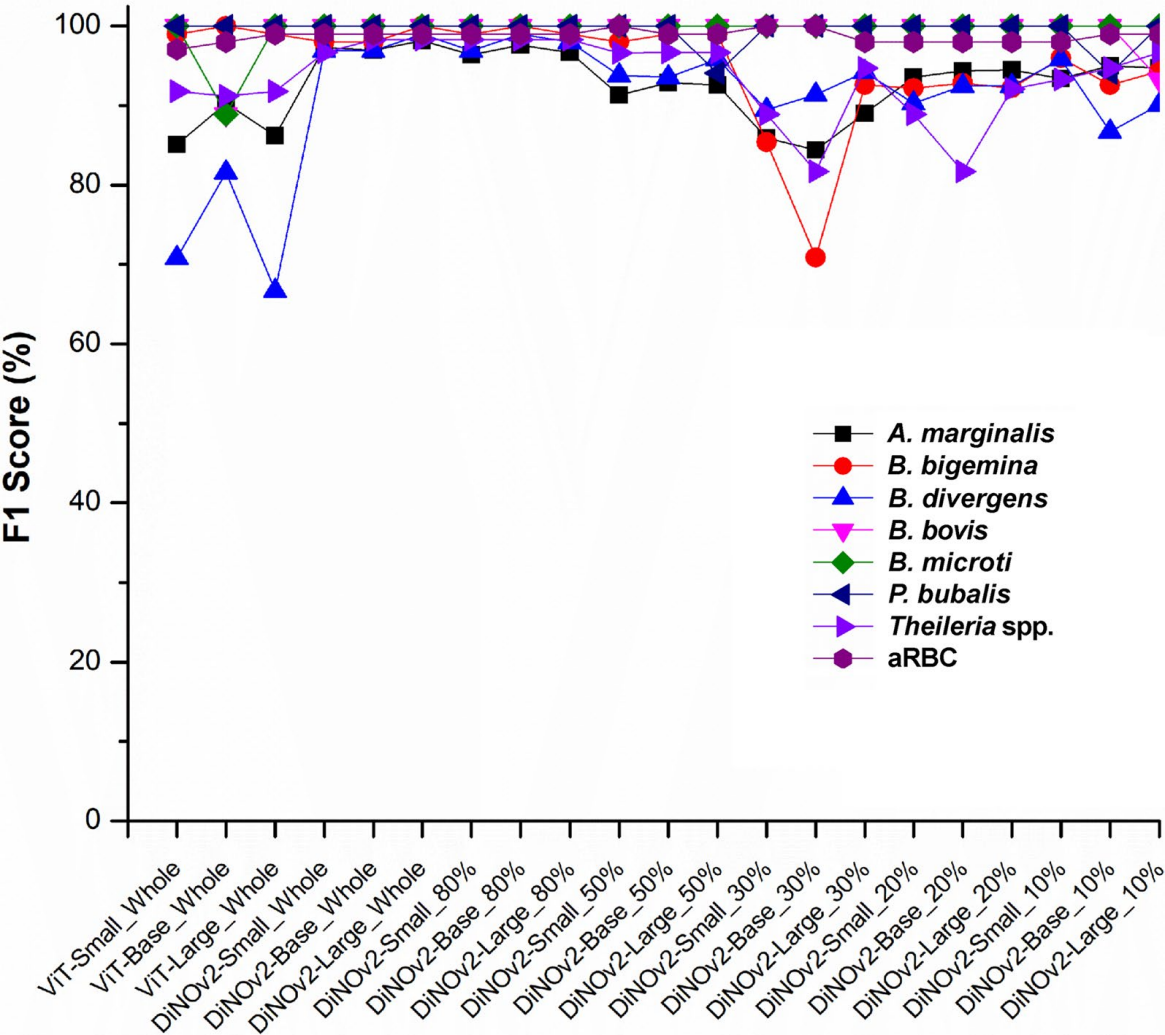
Harmonic mean as the F1 score. The baseline models used are the Small, Base, and Large versions of the Vision Transformer, while the SSL models are the Small, Base, and Large versions of DiNOv2. A vertical dashed line separates the two sides: the left side represents models trained on the full dataset (both SL and SSL), and the right side shows the performance of SSL models trained on data partitions

To address fluctuations in recall and precision, we evaluated the F1 score as a balanced measure of these metrics (Fig. 7). SSL models trained on complete datasets achieved substantially higher F1 scores than SL models. Notably, SSL models maintained consistent performance between full datasets and 80% partitions, demonstrating their robustness even with moderately reduced training data.

The F1 score demonstrated consistent stability for *B. microti*, *P. bubalis*, *B. bovis*, and aRBC across all data partitions. However, remaining classes showed performance fluctuations, particularly between 50% and 10% data partitions. Crucially, the DiNOv2-Large model maintained superior performance compared to other SSL variants at equivalent training sample sizes. Most significantly, when trained with just 20% of data, the DiNOv2-Large model exhibited exceptional stability across varying dataset sizes and compositions, underscoring its remarkable robustness in low-data scenarios.

### Generalization across thresholds of selected-trained models

**Fig. 8:**
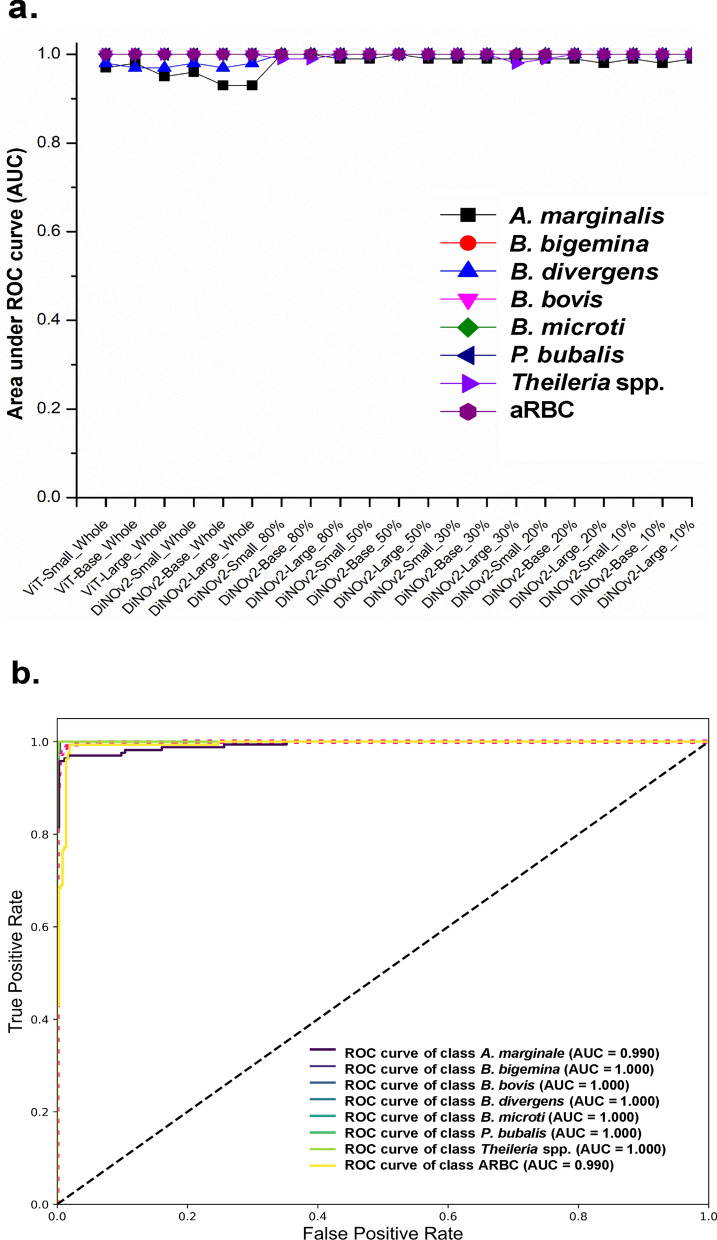
Area under the ROC curve. The baseline models used are the Small, Base, and Large versions of the Vision Transformer, while the SSL models are the Small, Base, and Large versions of DiNOv2. A vertical dashed line separates the two sides: the left side represents models trained on the full dataset (both SL and SSL), and the right side shows the performance of SSL models trained on data partitions

We assessed the generalization capability of selected SSL models using ROC curve analysis, a robust approach for evaluating imbalanced class distributions. For models trained on the full dataset, AUC values were consistently high (Fig. 8a). While SSL models showed marginal performance decreases, the *A. marginale* class maintained an exceptional AUC > 0.930, with all other classes achieving near-perfect scores (0.970–1.000).

Remarkably, five classes—*B. bigemina*, *B. divergens*, *B. microti*, *Theileria* spp., and aRBC—attained flawless classification despite severe dataset imbalance and variability. These results demonstrate the exceptional robustness of DiNOv2 architectures in accurately discriminating all eight classes, even under challenging imbalanced conditions (Fig. 8b).

## Discussion

This study successfully identified eight classes of clinically significant pathogens, including human- and animal-infecting *Babesia* species, water buffalo malaria parasites (*P. bubalis*), veterinary-relevant bacteria (*A. marginale*), and animal red blood cells (aRBC) using the semi-supervised DiNOv2 model [31]. Model development involved careful processing of microscopic images (1000× magnification) through the Segment Anything Model (SAM) algorithm [20] for semantic segmentation, followed by class labels. The optimal models were selected based on plateauing training accuracy and loss curves (Fig. 3), indicating convergence.

Comparative analysis revealed closely matched performance metrics between supervised learning (SL) and self-supervised learning (SSL) models when trained on full datasets, with ViT models achieving > 96% accuracy and DiNOv2 models exceeding 99% accuracy (Fig. 4). The model’s 99% overall accuracy is inflated by the dominant RBC class. A more informative metric is the 26.3% misclassification rate for minority classes, which better reflects the model’s performance on rare samples. This relationship between high-level metrics and per-class error rates is detailed in Supplementary Tables S1–S3. These findings align with prior research demonstrating that SSL pretraining surpasses ImageNet-based pretraining for non-medical images [12, 30], underscoring how pretraining strategy critically influences medical imaging diagnostic accuracy. The DINOv2 approach has similarly outperformed state-of-the-art models in endoscopic depth estimation tasks [15]. For specificity and F1 scores (the harmonic means of recall and precision), SL and SSL models showed comparable results (86% versus 85%, respectively). While the large ViT variant matched SSL’s peak specificity (98.3%), SSL models demonstrated greater stability (98.2–98.5%) compared with SL’s wider variability (91.3–99%). These results highlight the effectiveness of both SL and SSL approaches for learning features from segmented blood cell images[11, 14], with SSL offering more consistent performance.

To evaluate the impact of reduced training data on feature learning, SSL models trained on partial datasets (80%–10%) were compared with SL models trained on full data. Specificity and accuracy metrics indicated that most models effectively identified true negatives (TN), with the exception of *A. marginale* (Fig. 5a–b). The observed misclassification rates further supported these findings, revealing fluctuations in performance—particularly for *A. marginale* and both human- and animal-infecting *Babesia* species (Fig. 5c). Notably, the most pronounced instability occurred when SSL models were trained with only 10% of the data, suggesting a practical lower limit of 20% for reliable model training. While precision remained stable across most classes, *A. marginale* exhibited a marked decline. Even models trained on full datasets displayed sporadic inconsistencies for *B. divergens*, *B. bovis*, and *B. microti* (Fig. 6), likely attributable to imbalanced sample sizes and inherent data variability. Prior studies have demonstrated that state-of-the-art performance can achieve a 9–17 percentage-point accuracy improvement with as few as 100 labeled samples per class (representing 1%–10% of full data) across diverse public datasets [32]. Although this study identifies 20% of full data as optimal, seven classes contained fewer than 100 labeled training images. These findings underscore the importance of dataset characteristics and the selection of appropriate pretraining strategies for specific downstream tasks [12, 30]. For a complete performance picture, both macro- and micro-averages should be reported. The macro-average ensures fairness across all classes, which is critical for detecting rare diseases, while the micro-average reflects overall accuracy, favoring larger classes. Experiments revealed a critical threshold: models trained with less than 20% of the data suffered a systemic collapse, showing deceptively high micro-averages but failing on minority classes (e.g., *P. bulbalis* F1 scores of 12–18%). Using 20% of the data proved to be a stable threshold, yielding consistent precision between 93% and 96% and balancing the precision-recall trade-off (Supplementary Tables S4–S6).

The study faced limitations due to a highly imbalanced fine-tuning dataset comprising three distinct size categories: (i) *A. marginale* and red blood cells (800–1000 cells/class), (ii) animal-infecting *Babesia* and *Theileria* species (100–150 cells/class), and (iii) human-infecting *Babesia* species (10–20 cells/class). While this class imbalance negatively affected all SL model variants—particularly evident in *B. divergens* (F1 score < 80%)—SSL models demonstrated superior robustness, consistently achieving F1 scores > 90% (Fig. 6) [33]. Notably, the data imbalance still impacted feature learning in smaller SSL architectures (base and small versions), causing performance fluctuations. Fortunately, the DiNOv2-Large variant effectively mitigated these issues, demonstrating both stable performance and improved F1 scores compared with its smaller counterparts. These findings suggest that large SSL models possess enhanced capability to handle imbalanced datasets while maintaining high performance with limited labeled data [34].

An additional limitation stemmed from inherent biological variability in cell morphology and technical inconsistencies in slide preparation. These included variations in staining quality (dependent on both staining protocols and technician skill) and inter-observer interpretation differences, all of which impacted dataset homogeneity [11]. While these factors represent postprocessing variables introduced prior to microscopic observation, data augmentation techniques combined with the inherent robustness of SSL models effectively mitigated these challenges [33]. The strong generalization capability of the optimized SSL models was further corroborated by their high AUC-ROC performance (Fig. 8a, b) [34]. Nevertheless, accurate interpretation of cellular morphology remains an active area of research with persistent challenges [33]. The model demonstrated remarkable and consistent performance across multiple randomized experiments, confirming its potential for clinical deployment. It achieved outstanding accuracy (≥ 98.5%) and excellent specificity (≥ 98.9%), with strong recall and precision (≥ 96%) underscoring its balanced discriminative power. This is complemented by high PR-AUC scores, indicating robust performance on imbalanced data, and well-calibrated predictions where confidence scores reliably reflect actual accuracy (Supplementary Table S7).

The study contributions are enhancing healthcare SSL via unlabeled data, showing SSL outperforms supervised learning on imbalanced segmentation without augmentation, and demonstrating effective transfer to unseen datasets after fine-tuning. These advantages can be delivered to multiple, remote domains via a telehealth system, using mobile and web applications to compensate for a lack of expensive apparatus.

In conclusion, this study demonstrates the potential of DiNOv2 models trained on partial datasets to serve as cost-effective diagnostic tools in resource-limited settings. By reducing reliance on expensive equipment, our approach offers practical solutions for both healthcare laboratories and field surveillance operations. The findings support further development of these models as automated screening tools for human and veterinary medicine, potentially transforming disease detection capabilities in underserved areas.

## Supplementary Information

Supplementary Material 1.

Supplementary Material 2.

Supplementary Material 3.

## Data Availability

The data supporting the findings of this study are available in the repository at URL: https://gitlab.com/veerayuth.ki/intelligent-identification-of-medical-and-veterinary-intracellular-protozoa-by-using-self-supervised-learning/-/blob/eb6324a9a7d299dc41833682b4d760bd99d1187a/dataset.zip
