# Supplementary material for: Intelligent identification of medical and veterinary intracellular protozoa by using self-supervised learning
Source: Parasit Vectors. 2026 Feb 9;19:98. doi: 10.1186/s13071-026-07257-9 (PMC12937529; doi:10.1186/s13071-026-07257-9)
Supplement: Supplementary file 3 — Supplementary Material 3. [file 13071_2026_7257_MOESM3_ESM.docx]

**Table S3. Model AUC versus misclassification rate.** A comparison of baseline Vision Transformer (ViT) and self-supervised (DINOv2) models, across small, base, and large sizes, trained on the full- and partition datasets. (Accu: Accuracy, Misc: Misclassification Rate).

| **Model version** | **Data partition** | **Class name** | | | | | | | | | | | | | | | | |
| --- | --- | --- | --- | --- | --- | --- | --- | --- | --- | --- | --- | --- | --- | --- | --- | --- | --- | --- |
|  |  | ***A. marginale*** | | ***B. bigemina*** | | ***B. bovis*** | | ***B. divergens*** | | ***B. microftiCDC*** | | ***P. bulbalis*** | | ***Theileria spp.*** | | **aRBC** | |  |
|  |  | ***AUC*** | ***Misc*** | ***AUC*** | ***Misc*** | ***AUC*** | ***Misc*** | ***AUC*** | ***Misc*** | ***AUC*** | ***Misc*** | ***AUC*** | ***Misc*** | ***AUC*** | ***Misc*** | ***AUC*** | ***Misc*** |  |
| ViT-Small | Whole | 0.970 | 0.084 | 1.000 | 0.002 | 0.980 | 0.073 | 1.000 | 0.000 | 1.000 | 0.000 | 1.000 | 0.000 | 1.000 | 0.010 | 1.000 | 0.006 |  |
| ViT-Base | Whole | 0.980 | 0.059 | 1.000 | 0.000 | 0.970 | 0.036 | 1.000 | 0.004 | 1.000 | 0.004 | 1.000 | 0.000 | 1.000 | 0.010 | 1.000 | 0.004 |  |
| ViT-Large | Whole | 0.950 | 0.080 | 1.000 | 0.002 | 0.970 | 0.086 | 1.000 | 0.000 | 1.000 | 0.000 | 1.000 | 0.000 | 1.000 | 0.010 | 1.000 | 0.002 |  |
| DiNOv2-Small | Whole | 0.960 | 0.084 | 1.000 | 0.000 | 0.980 | 0.069 | 1.000 | 0.000 | 1.000 | 0.000 | 1.000 | 0.000 | 1.000 | 0.008 | 1.000 | 0.002 |  |
| DiNOv2-Base | Whole | 0.930 | 0.086 | 1.000 | 0.000 | 0.970 | 0.079 | 1.000 | 0.000 | 1.000 | 0.010 | 1.000 | 0.000 | 1.000 | 0.006 | 1.000 | 0.004 |  |
| DiNOv2-Large | Whole | 0.930 | 0.025 | 1.000 | 0.002 | 0.980 | 0.004 | 1.000 | 0.000 | 1.000 | 0.000 | 1.000 | 0.000 | 1.000 | 0.002 | 1.000 | 0.002 |  |
| DiNOv2-Small | 80% | 1.000 | 0.019 | 1.000 | 0.002 | 1.000 | 0.004 | 1.000 | 0.000 | 1.000 | 0.000 | 1.000 | 0.000 | 0.990 | 0.002 | 1.000 | 0.002 |  |
| DiNOv2-Base | 80% | 1.000 | 0.023 | 1.000 | 0.002 | 1.000 | 0.006 | 1.000 | 0.000 | 1.000 | 0.000 | 1.000 | 0.000 | 0.990 | 0.002 | 1.000 | 0.002 |  |
| DiNOv2-Large | 80% | 0.990 | 0.015 | 1.000 | 0.000 | 1.000 | 0.002 | 1.000 | 0.000 | 1.000 | 0.000 | 1.000 | 0.000 | 1.000 | 0.002 | 1.000 | 0.002 |  |
| DiNOv2-Small | 50% | 0.990 | 0.059 | 1.000 | 0.004 | 1.000 | 0.011 | 1.000 | 0.000 | 1.000 | 0.000 | 1.000 | 0.000 | 1.000 | 0.004 | 1.000 | 0.000 |  |
| DiNOv2-Base | 50% | 1.000 | 0.048 | 1.000 | 0.002 | 1.000 | 0.011 | 1.000 | 0.000 | 1.000 | 0.000 | 1.000 | 0.000 | 1.000 | 0.004 | 1.000 | 0.002 |  |
| DiNOv2-Large | 50% | 0.990 | 0.050 | 1.000 | 0.002 | 1.000 | 0.008 | 1.000 | 0.000 | 1.000 | 0.000 | 1.000 | 0.002 | 1.000 | 0.004 | 1.000 | 0.002 |  |
| DiNOv2-Small | 30% | 0.990 | 0.033 | 1.000 | 0.002 | 1.000 | 0.008 | 1.000 | 0.000 | 1.000 | 0.000 | 1.000 | 0.000 | 1.000 | 0.006 | 1.000 | 0.002 |  |
| DiNOv2-Base | 30% | 0.990 | 0.063 | 1.000 | 0.013 | 1.000 | 0.011 | 1.000 | 0.000 | 1.000 | 0.000 | 1.000 | 0.000 | 1.000 | 0.006 | 1.000 | 0.004 |  |
| DiNOv2-Large | 30% | 0.990 | 0.075 | 1.000 | 0.021 | 1.000 | 0.023 | 1.000 | 0.000 | 1.000 | 0.000 | 1.000 | 0.000 | 0.980 | 0.002 | 1.000 | 0.002 |  |
| DiNOv2-Small | 20% | 0.990 | 0.044 | 1.000 | 0.002 | 1.000 | 0.006 | 1.000 | 0.000 | 1.000 | 0.000 | 1.000 | 0.000 | 0.990 | 0.006 | 1.000 | 0.002 |  |
| DiNOv2-Base | 20% | 0.990 | 0.096 | 1.000 | 0.004 | 1.000 | 0.008 | 1.000 | 0.000 | 1.000 | 0.000 | 1.000 | 0.000 | 1.000 | 0.006 | 1.000 | 0.002 |  |
| DiNOv2-Large | 20% | 0.980 | 0.042 | 1.000 | 0.015 | 1.000 | 0.017 | 1.000 | 0.000 | 1.000 | 0.000 | 1.000 | 0.000 | 1.000 | 0.013 | 1.000 | 0.004 |  |
| DiNOv2-Small | 10% | 0.990 | 0.036 | 1.000 | 0.013 | 1.000 | 0.013 | 1.000 | 0.000 | 1.000 | 0.000 | 1.000 | 0.000 | 1.000 | 0.025 | 1.000 | 0.004 |  |
| DiNOv2-Base | 10% | 0.980 | 0.036 | 1.000 | 0.015 | 1.000 | 0.013 | 1.000 | 0.000 | 1.000 | 0.000 | 1.000 | 0.000 | 1.000 | 0.010 | 1.000 | 0.004 |  |
| DiNOv2-Large | 10% | 0.990 | 0.044 | 1.000 | 0.008 | 1.000 | 0.008 | 1.000 | 0.002 | 1.000 | 0.000 | 1.000 | 0.000 | 1.000 | 0.008 | 1.000 | 0.004 |  |
